# Protective Effect of Omega-3 Fatty Acids in Fish Consumption Against Breast Cancer in Asian Patients: A Meta-Analysis

**DOI:** 10.31557/APJCP.2019.20.2.327

**Published:** 2019

**Authors:** Ricvan Dana Nindrea, Teguh Aryandono, Lutfan Lazuardi, Iwan Dwiprahasto

**Affiliations:** 1 *1Doctoral Program,*; 3 *Department of Surgery, *; 4 *Department of Health Policy and Management,*; 5 *Department of Pharmacology and Therapy, Faculty of Medicine, Public Health and Nursing, Universitas Gadjah Mada, Yogyakarta City,*; 2 *Department of Public Health, Faculty of Medicine, Universitas Andalas, Padang City, Indonesia. *

**Keywords:** Breast neoplasms, fatty acids, omega-3, fish consumption

## Abstract

**Objective:**

This systematic review and meta-analysis was performed to determine the protective effect of omega-3 fatty acids in fish consumption against breast cancer in Asian patients.

**Methods:**

The authors conducted a meta-analysis of published research articles on protective effect of omega-3 fatty acids in fish consumption against breast cancer in Asian patients published between January 2000 and July 2018 in online database of PubMed, ProQuest and EBSCO. Pooled odds ratios (OR) were calculated by using fixed and random-effect models. Publication bias was visually evaluated by using funnel plots and statistically assessed in Egger’s and Begg’s tests. Data were processed by Review Manager 5.3 (RevMan 5.3) and Stata version 14.2 (Stata Corporation).

**Results:**

This study reviewed 913 articles. There were 11 studies which conducted systematic review then continued by meta-analysis of relevant data with total number of samples were 130,365 patients. The results showed there was protective effect of omega-3 fatty acids in fish consumption against breast cancer in Asian patients (OR = 0.80 [95% CI 0.73-0.87, p <0.00001]). There was not any study with significant publication bias included.

**Conclusion:**

This analysis confirmed the protective effect of omega-3 fatty acids in fish consumption against breast cancer in Asian patients.

## Introduction

Breast cancer is a major health concern in the world as it is one of the most common malignancies found worldwide. Although Asian countries has lower incidence rate than Europe and United States, the death rate of breast cancer in Asia is considerable (Jemal et al., 2011). This kind of neoplasm has multifactorial etiology. The breast cancer risk factors are divided into nonmodifiable and modifiable risk factors (Nindrea et al., 2017; Nindrea et al., 2018). In the past few decades, epidemiological studies have suggested that healthy diet and lifestyle are essential to prevent breast cancer (Lambrechts et al., 2011; Gao et al., 2013). One of the dietary factors associated with breast cancer risk is low intake of fish (Nindrea et al., 2017).

The low intake of fish is associated with higher breast cancer risk. Some studies have suggested that omega-3 fatty acids in fish consumption is beneficial for breast cancer prevention and survivorship. Fish fatty acid is commonly referred to longer chain omega-3 polyunsaturated fatty acids (PUFAs), eicosapentaenoic acid (EPA) and docosahexaenoic acid (DHA) (Kim et al., 2009; Fabian et al., 2015). However, due to wide geographical variation, the demography of breast cancer patients in developing countries differs from those in developed countries. The changes in dietary pattern and lifestyle due to globalization and economic improvement, as well as the availability of breast cancer screening programme might affect the breast cancer risk of the people. 

A study by Zheng et al., (2013) firstly found that fish consumption is associated with lower risk of breast cancer. High intake of fatty fish is significantly associated with lower breast cancer risk (Gao et al., 2014). However, some subsequent studies of fish consumption and breast cancer led to contradictory conclusions (Stripp et al., 2003; Holmes et al., 2003; Engeset et al., 2006). So, in fact, the research findings seem to be inconsistent. The different results possibly happened due to different dietary pattern of fish in Asian population and Western population, whom the latter has lower intake, which may impact the significance of the results.

Through measurement of breast cancer risk, it can be classified whether a person has a safe risk to breast cancer, being adequate for breast cancer prevention or harmful to the occurrence of breast cancer (Nindrea et al., 2018). Therefore, this study aims to determine the protective effect of omega-3 fatty acids in fish consumption against breast cancer in Asian patients through meta-analysis study, so that the conclusion drawn from studies on omega-3 fatty acids in fish consumption and breast cancer association will have stronger strength of recommendation.

## Materials and Methods

*Study design and research sample*

This study was a quantitative research with meta-analysis study design. The meta-analysis of the study followed PRISMA Statement to prefer reporting items (Liberati et al., 2009). Meta-analysis was performed to examine protective effect of omega-3 fatty acids in fish consumption against breast cancer in Asian patients. The research samples were published research articles published between January 2000 and July 2018 in online database of PubMed, ProQuest and EBSCO. 

*Operational definitions*

The variables of this study included omega-3 fatty acids in fish consumption as independent variable and breast cancer as dependent variable.

*Research procedure*

**Figure 1:**
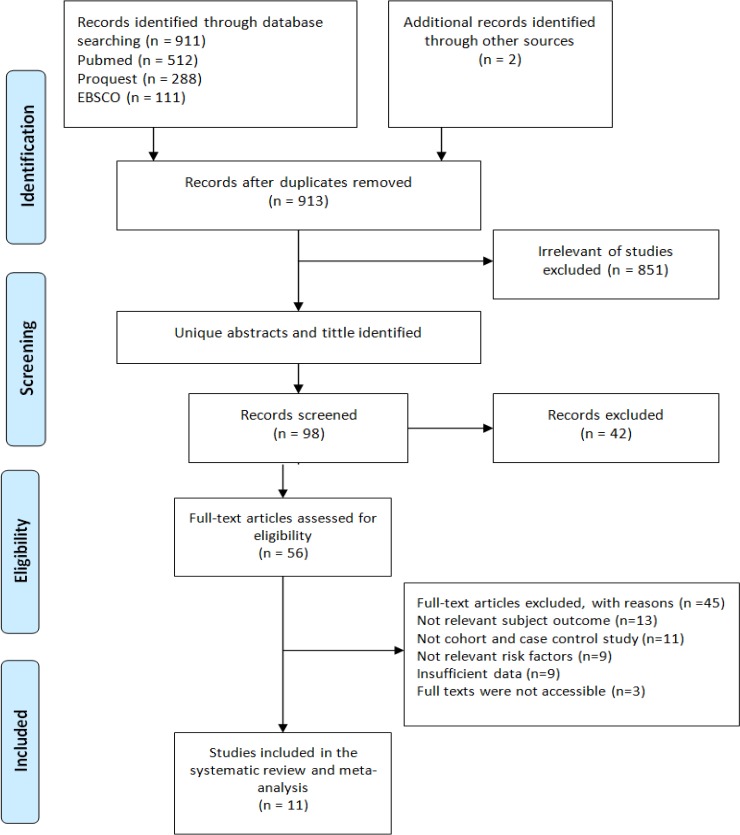
Flow Diagram Research Procedure

This research was conducted through data collection to identify published research articles on protective effect of omega-3 fatty acids in fish consumption against breast cancer in Asian patients in online database of PubMed, ProQuest and EBSCO (Figure 1). 

Identification process of 913 articles was held by identifying the title of the articles, continued by reviewing the abstract, and then the full-text of the articles. An article must be excluded if: (a) unrelevant to subject outcome, (b) the methods was not case control and cohort study (c) the information provided in the results was insufficient for data extraction.

*Data collection technique*

The data collection was performed by online searching. The online searching was limited to English language articles. The article type was limited to journal articles. The research subject was limited to research with human subject. The time of publication was limited from January 2000 to July 2018. The abstract of articles with potentially relevant title were reviewed, while the articles with irrelevant title were excluded. Furthermore, articles that have potentially relevant abstract were continue to be reviewed in full-text, while the articles with irrelevant abstract were excluded. The inclusion criteria of this study sample was research on protective effect of omega-3 fatty acids in fish consumption against breast cancer in Asian patients with case control or cohort study methods. Exclusion criteria were (1) the article was not available in full-text and (2) the inclusion criteria were not satisfyingly fulfilled or the information provided in the article was insufficient for data extraction. The following data were obtained from each article: first name of authors and year of publication, region, type of study, number of sample, pre or postmenopause status of the sample, fish consumption and dietary assessment.

Two independent investigators carefully extracted information from all studies that fulfilled the inclusion criteria in accordance with a standardized protocol. Disagreements were resolved by three other investigators. Quality assessment was conducted by using Newcastle–Ottawa Quality Assessment Scale (NOS). The papers with a total score of 0-3, 4-6, and 7-9 points were classified as the poor, moderate, and high quality (Wells et al., 2009).

*Data analysis*

The analysis was performed to obtain the value of log odds ratio as the combined odds ratio value from the research. Results were pooled using odds ratio with corresponding 95% confidence intervals (CIs). Significant heterogeneity was indicated by I^2^>50% because these tests presented minimal statistical power on cases with few studies and small sample sizes. A random effect model was used when significant heterogeneity was observed; otherwise, a fixed effect model was used. Publication bias was visually evaluated by using funnel plots and statistically assessed through Egger’s and Begg’s tests. Meta-analysis was carried out in Stata version 14.2 (Stata Corporation). A two-tailed P-value of <0.05 was considered as statistically significant.

**Table 1:** Systematic Review Dietary Omega-3 Fatty Acids in Fish Consumption Protective Againts Breast Cancer Patients in Asia

First author, year	Region	Type of study	Number of sample		Pre-or-Post menopause	Fish consumption	Dietary assesment	Effect size	NOS
			Cases	Controls					
Yaw et al., 2014	Malaysia	Cohort	368	368	Post menopause	3.21 gr/day	Food frequency questionnaire	RR (95% CI): 0.81(0.68-0.97)	7
Sangrajrang et al., 2013	Thailand	Case control	1,130	1,142	Both	>340 gr/ week vs < 76 gr/ week	Food frequency questionnaire	OR (95% CI): 0.9(0.65-1.23)	8
Murff et al., 2011	China	Cohort	712	71,859	Both	51.1 gr/ day vs 50.0 gr/ day	Food frequency questionnaire	RR (95% CI): 0.95(0.75-1.20)	8
Zhang et al., 2009	China	Case control	438	438	Both	87.85 gr/ day vs 18.62 gr/ day	Food frequency questionnaire	OR (95% CI): 0.56(0.37-0.85)	7
Kim et al., 2009	South Korea	Case control	360	358	Both	24.1 gr/ day vs 21.8 gr/day	Food frequency questionnaire	OR (95% CI): 0.62(0.41-0.93)	7
Kuriki et al., 2007	Japan	Case control	103	309	Both	22.2 gr/ 1,000 kcal vs 20.3 gr/ 1,000 kcal	Food frequency questionnaire	OR (95% CI): 0.59(0.31-1.12)	7
Shannon et al., 2005	China	Case control	378	1,070	Both	≥ 4.3 gr/ week vs < 1.3 gr/ week	Food frequency questionnaire	OR (95% CI): 1.36(0.85-2.18)	7
Gago-Dominguez et al., 2003	Singapore	Cohort	314	314	Both	80.5 gr/ day vs 24.5 gr/ week	Food frequency questionnaire	RR (95% CI): 0.74(0.59-0.94)	7
Hirose et al., 2003	Japan	Case control	2,385	19,013	Both	3–4 times/week vs < 1–3 times/ week	Food frequency questionnaire	OR (95% CI): 0.95(0.71-1.28)	8
Wakai et al., 2005	Japan	Cohort	13,145	13,146	Both	≥ 0.63% of energy vs < 0.29% of energy	Food frequency questionnaire	RR (95% CI): 0.50(0.25-1.00)	8
Dai et al., 2002	China	Case control	1,459	1,556	Both	12.4 gr/day vs 9.7 gr/ day	Food frequency questionnaire	OR (95% CI): 0.68(0.54-0.86)	8
Number of samples			20,792	109,573					

NOS, Newcastle; Ottawa Quality Assessment Scale

**Figure 2:**
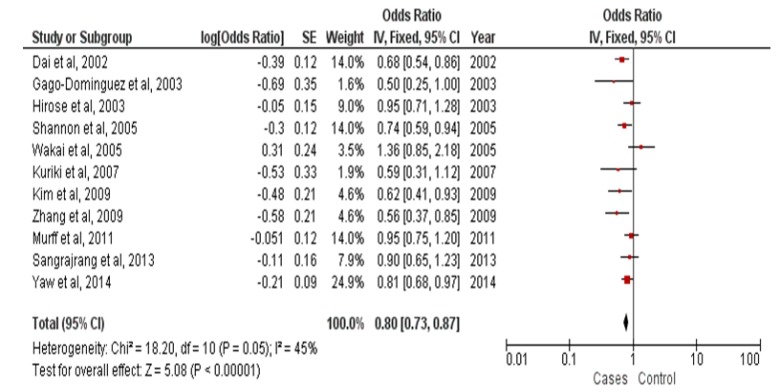
Forest Plots Dietary Omega-3 Fatty Acids in Fish Consumption Protective Againts Breast Cancer Patients in Asia

**Figure 3:**
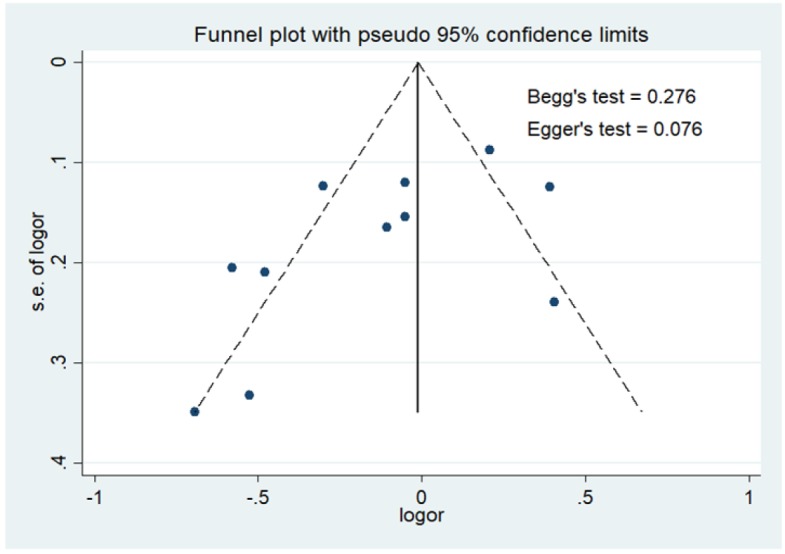
Funnel Plot to Examine Publication Bias for Dietary Omega-3 Fatty Acids in Fish Consumption Protective Againts Breast Cancer Patients in Asia

## Results

The selection of articles was conducted to obtain 11 studies with total samples of 130,365 patients related to protective effect of omega-3 fatty acids in fish consumption against breast cancer in Asian patients (Table 1).

Figure 2 showed meta-analysis study on protective effect of omega-3 fatty acids in fish consumption against breast cancer in Asian patients (OR = 0.80 [95% CI 0.73-0.87, p<0.00001]). Heterogeneity in studies on family history of breast cancer and breast cancer risk in Malays ethnicity in Malaysia and Indonesia (Pheterogeneity = 0.05; I^2^= 45%) showed a variation of homogeneous research on the occurrence of breast cancer. Funnel plots to identify publication bias among studies protective effect of omega-3 fatty acids in fish consumption against breast cancer in Asian patients was shown in Figure 3.

In Figure 3, it was shown that there was not significant publication bias for studies on protective effect of omega-3 fatty acids in fish consumption against breast cancer in Asian patients included with Egger’s test (P = 0.076) and Begg’s test (P = 0.276).

## Discussion

This analysis confirmed the protective effect of omega-3 fatty acids in fish consumption against breast cancer in Asian patients (OR = 0.80 [95% CI 0.73-0.87, p <0.00001]). Dietary fish had been studied in a variation of homogeneous research or no heterogeneity among studies on the occurrence of breast cancer. 

High consumption of fish has been associated with lower breast cancer risk (Zheng et al., 2013; Gao et al., 2014). On the other hand, some studies had led to contrary results which fish intake had no correlation to lower risk of breast cancer (Stripp et al., 2003; Holmes et al., 2003; Engeset et al., 2006). In other population, such as European, fish intake had been positively associated with lower breast cancer risk (OR=1.11) as well as in Western countries (OR=1.08) and US (OR=1.05) (Zheng et al., 2013). In Norway, no association found between 5.54 g to 96.77g/ day of fish consumption and breast cancer development (Engeset et al., 2006). The contradictory results of our study to the latter study, possibly due to the different habit of fish consumption in Asian people as the subjects of our study. It has been known that fish intake of Asian population is considerably higher than Western population (Terry et al., 2003; Fabian et al., 2015). Therefore, fish intake in Western populations might be too low to affect breast cancer risk as well as to give an expected protective effect.

Previous study with meta-analysis which combined 26 international studies involving 883,585 female participants, including 20,000 breast cancer patients, found that omega-3 fatty acids intake from fish provided 14% reduction of breast cancer risk (Zheng et al., 2013). Another study by Yang et al., (2014), has found that by increasing the ratio of omega-3 and omega 6 intake, lower risk of breast cancer can be obtained. The study also suggested higher ratio of omega-3 and omega 6 intake as deterrent factor of breast cancer.

Fish is well known as an important source of protein worldwide. Commonly found fish has omega-3 as its content. Omega-3 has been known as fat content of common fish (FAO, 2014). Fish fatty acids usually referred to the longer chain of omega-3 polyunsaturated fatty acids (PUFAs), eicosapentaenoic acid (EPA) and docosahexaenoic acid (DHA) (Kim et al., 2009; Fabian et al., 2015). EPA and DHA are expected to prevent breast cancer by decreasing epidermal growth factor receptor and human epidermal growth factor-2 signaling which reduce proliferation. Ki-67 decrease had also been observed in benign and malignant mammary neoplasm after EPA and DHA supplementation in most preclinical models (Jiang et al., 2012; Yee et al., 2013; Harahap et al., 2018). In addition, factor-κB nuclear translocation and signaling also reduced through the agonist effects of EPA and DHA on peroxisome proliferator-activated receptor gamma and through interaction with the G protein receptor GPR120, which are expected to decrease inhibitors of apoptosis as well as cytokines, adhesion molecules, and metalloproteases (Calder, 2013). 

There were several limitations in this meta-analysis review. First, three studies seemed potentially eligible to be included in this meta-analysis but the full-texts were not accessible. Second, nine studies seemed potentially eligible to be included in this meta-analysis but they had insufficient data and unrelevant risk factors for calculation. This issue may raise the possibility of selection bias. Third, data validation not available for dietary questionaires in several studies. Fourth, the number of cases sample in one study was relatively small as it can reduce the statistical strength (Kuriki et al., 2007).

This analysis confirmed the protective effect of omega-3 fatty acids in fish consumption against breast cancer in Asian patients. Based on fish consumption, Asian population has more significant protection from breast cancer than Western population. The results of this study recommend the need to maintain dietary fish intake. Dietary fish could possibly effective for breast cancer prevention. This study also suggests the need for education and counseling about eating habits and the importance of consuming foods with high fish content. The example of fish which commonly found and contain omega-3 fats are salmon, mackerel, herring, lake trout, bluefin tuna, sturgeon, sablefish, anchovy, albacore tuna, whitefish, arctic char, sardines, bluefish, mullet, halibut, striped bass, mahi mahi, pollock, rockfish, rainbow trout, shark, catfish, carp, cod, flounder, grouper, haddock, ocean perch, red snapper, swordfish, pike, sole, tilapia, etc. 

## Conflict of interest

The authors declare no conﬂict of interest.
